# A novel, integrated approach for understanding and investigating Healthcare Associated Infections: A risk factors constellation analysis

**DOI:** 10.1371/journal.pone.0282019

**Published:** 2023-03-24

**Authors:** Mariachiara Carestia, Massimo Andreoni, Ersilia Buonomo, Fausto Ciccacci, Luigi De Angelis, Gerardo De Carolis, Patrizia De Filippis, Daniele Di Giovanni, Leonardo Emberti Gialloreti, Carla Fontana, Luca Guarente, Andrea Magrini, Marco Mattei, Stefania Moramarco, Laura Morciano, Claudia Mosconi, Stefano Orlando, Giuseppe Quintavalle, Fabio Riccardi, Viviana Santoro, Leonardo Palombi

**Affiliations:** 1 Department of Biomedicine and Prevention, University of Rome Tor Vergata, Rome, Italy; 2 Infectious Diseases Clinic, University Hospital “Tor Vergata”, Rome, Italy; 3 Department of Systems Medicine, University of Rome “Tor Vergata”, Rome, Italy; 4 Unicamillus, Saint Camillus International University of Health Sciences, Rome, Italy; 5 Health Management, University Hospital “Tor Vergata”, Rome, Italy; 6 Industrial Engineering Department, University of Rome ‘Tor Vergata’, Rome, Italy; 7 UOC Microbiology and Bio Bank National Institute of Infectious Diseases “Lazzaro Spallanzani”, Rome, Italy; 8 Department of Prevention, Local Health Authority—Rome 6, Service of Hygiene and Public Health, Rome, Italy; 9 General Direction, University Hospital “Tor Vergata”, Rome, Italy; Gabriele d’Annunzio University of Chieti and Pescara: Universita degli Studi Gabriele d’Annunzio Chieti Pescara, ITALY

## Abstract

**Introduction:**

Healthcare-associated infections (HAIs) and antimicrobial resistance (AMR) are major public health threats in upper- and lower-middle-income countries. Electronic health records (EHRs) are an invaluable source of data for achieving different goals, including the early detection of HAIs and AMR clusters within healthcare settings; evaluation of attributable incidence, mortality, and disability-adjusted life years (DALYs); and implementation of governance policies. In Italy, the burden of HAIs is estimated to be 702.53 DALYs per 100,000 population, which has the same magnitude as the burden of ischemic heart disease. However, data in EHRs are usually not homogeneous, not properly linked and engineered, or not easily compared with other data. Moreover, without a proper epidemiological approach, the relevant information may not be detected. In this retrospective observational study, we established and engineered a new management system on the basis of the integration of microbiology laboratory data from the university hospital “Policlinico Tor Vergata” (PTV) in Italy with hospital discharge forms (HDFs) and clinical record data. All data are currently available in separate EHRs. We propose an original approach for monitoring alert microorganisms and for consequently estimating HAIs for the entire period of 2018.

**Methods:**

Data extraction was performed by analyzing HDFs in the databases of the Hospital Information System. Data were compiled using the AREAS-ADT information system and ICD-9-CM codes. Quantitative and qualitative variables and diagnostic-related groups were produced by processing the resulting integrated databases. The results of research requests for HAI microorganisms and AMR profiles sent by the departments of PTV from 01/01/2018 to 31/12/2018 and the date of collection were extracted from the database of the Complex Operational Unit of Microbiology and then integrated.

**Results:**

We were able to provide a complete and richly detailed profile of the estimated HAIs and to correlate them with the information contained in the HDFs and those available from the microbiology laboratory. We also identified the infection profile of the investigated hospital and estimated the distribution of coinfections by two or more microorganisms of concern. Our data were consistent with those in the literature, particularly the increase in mortality, length of stay, and risk of death associated with infections with *Staphylococcus* spp, *Pseudomonas aeruginosa*, *Klebsiella pneumoniae*, *Clostridioides difficile*, *Candida* spp., and *Acinetobacter baumannii*. Even though less than 10% of the detected HAIs showed at least one infection caused by an antimicrobial resistant bacterium, the contribution of AMR to the overall risk of increased mortality was extremely high.

**Conclusions:**

The increasing availability of health data stored in EHRs represents a unique opportunity for the accurate identification of any factor that contributes to the diffusion of HAIs and AMR and for the prompt implementation of effective corrective measures. That said, artificial intelligence might be the future of health data analysis because it may allow for the early identification of patients who are more exposed to the risk of HAIs and for a more efficient monitoring of HAI sources and outbreaks. However, challenges concerning codification, integration, and standardization of health data recording and analysis still need to be addressed.

## Introduction

Hospital-acquired infections (HAIs) represent a major public health concern. In Europe, HAIs affect more than 90,000 patients in acute-care hospitals daily, thus resulting in approximately 4.5 million cases per year [1]. A study published in 2021 evaluated the incidence, attributable deaths, and burden of the most significant HAIs in Italy, which is estimated at 702.53 disability-adjusted life years (DALYs) per 100,000 population [2]. For comparison, in 2017 in Italy, the value of DALYs for ischemic heart disease, which is the major cause of death in the country and in the world, was 749 (697–805) DALYs per 100,000 population, thus confirming that HAIs represent a major public health issue.

Antimicrobial resistance (AMR) is a significant factor that affects HAIs. Data from the AR-ISS national antibiotic resistance surveillance system by the Italian National Institute of Health states that in 2019 in Italy, the resistance rates to the main classes of antibiotics for the eight alert microorganisms under surveillance (namely, *Staphylococcus aureus*, *Streptococcus pneumoniae*, *Enterococcus faecalis*, *Enterococcus faecium*, *Escherichia coli*, *Klebsiella pneumoniae*, *Pseudomonas aeruginosa*, and *Acinetobacter* species) were still high or even increasing compared with those of the previous years, [3, 4] thus placing Italy almost at the bottom of the league in Europe [5].

In 2018, 16,539 isolates of multidrug-resistant (MDR) *Escherichia coli*, which is by far the most frequently isolated species among the eight alert microorganisms, were reported in Italy. This number increased to 18,866 in 2019. Isolates of resistant *Staphylococcus aureus* were the second most represented species, with 8,581 isolates in 2018 and 9,939 in 2019, followed by *K*. *pneumoniae* with 5,913 isolates in 2018 and 7,782 isolates in 2019 [6].

With regard to the specific resistance profile, the resistance rate of *Escherichia coli* to third-generation cephalosporins remained stable (around 30%), whereas a decreasing trend over the last five years (2015–2019) was observed for the resistance rate of fluoroquinolones (from 44.4% in 2015 to 40.7% in 2019). Concerning carbapenem-resistant *K*. *pneumoniae*, an increase was observed from 26.8% in 2018 to 28.5% in 2019. However, by considering only laboratories that participated in the surveillance system in both 2018 and 2019, a decrease from 26.4% to 22.7% was observed; therefore, this result is linked to the higher number of data collected by a greater number of laboratories that joined the surveillance network in Italy. On the other hand, carbapenem resistance was confirmed to be very low for *Escherichia coli* (0.4%), showed a decrease in *P*. *aeruginosa* species (13.7%), and was stable with regard to *Acinetobacter* (79.2%). Among gram-negative bacteria, 36.2% of *K*. *pneumoniae* isolates in 2019 were MDR (resistant to third-generation cephalosporins, aminoglycosides, and fluoroquinolones). By contrast, only 11.7% of *Escherichia coli* were MDR.

For *P*. *aeruginosa*, in 2019 the percentage of resistance to three or more antibiotics, including piperacillin-tazobactam, ceftazidime, carbapenems, aminoglycosides and fluoroquinolones was 13.1%, a decrease compared to previous years. However, a high and increasing percentage of multi-resistance (fluoroquinolones, aminoglycosides and carbapenems) was observed (77.3%) for *Acinetobacter*. For *S*. *aureus*, the proportion of methicillin-resistant (MRSA) isolates remained stable (around 34%), while significant increases were seen in the proportion of vancomycin-resistant *Enterococcus faecium* isolates, which stood at 21.3% in 2019. Finally, For *P*. *aeruginosa* in 2019, the percentage of resistance to three or more antibiotics, including piperacillin–tazobactam, ceftazidime, carbapenems, aminoglycosides, and fluoroquinolones, was 13.1%, which is lower than the values in previous years. However, a high and increasing percentage of multiresistance (fluoroquinolones, aminoglycosides, and carbapenems) was observed for *Acinetobacter* (77.3%). For *Staphylococcus aureus*, the proportion of methicillin-resistant *Staphylococcus aureus* isolates remained stable (approximately 34%), whereas significant increases were observed in the proportion of vancomycin-resistant *Enterococcus faecium* isolates (21.3% in 2019). Finally, for *S*. *pneumoniae*, a slight increase was observed in both the proportion of penicillin- and erythromycin-resistant isolates (11.9% and 22.4%, respectively) [4].

Catheter-associated urinary tract infections (CAUTIs) are prevalent worldwide (900,000 cases/year in the United States), whereas hospital-acquired pneumonia and blood stream infections (BSIs) are deadly and account for 67% of US annual deaths associated with HAIs. Surgical site infections are the most common and costly HAIs for surgical patients [6].

The common variables associated with HAIs are (i) invasive devices, such as intubation or CAUTIs and BSIs with vascular devices [7]; (ii); (ii) patient characteristics and conditions, such as age, immunosuppression, or comorbidities [6, 8], and (iii) intensive care units (ICUs) and high-dependency units (HDUs) [7]. For instance, the SARS-CoV-2 outbreak in 2019 resulted in a high number of patients requiring intensive care, intubation, and antimicrobials and has led the scientific community to investigate the effect of this pathogen on HAIs. Many studies are now beginning to report an alarming increase in HAIs and AMR due to COVID-19 [9, 10], particularly if we consider the increased use of drugs, the prolonged time that many COVID-19 patients have to spend in hospitals, and the difficulties of implementing AMR stewardship programs correctly during this public health emergency. The current pandemic may soon lead to further increases in HAIs and AMR [11].

Several strategies must be adopted to reduce the effect of HAIs: (i) hand hygiene; (ii) maintaining a safe, clean, hygienic hospital environment; (iii) screening and categorizing patients into risk-stratified cohorts; (iv) following patient safety guidelines; (v) antibiotic stewardship; and (vi) public health surveillance [12]. Finally, surveillance data on HAIs can be used to assess the extent, escalation, and status of infections; examine, scan, and monitor the trends of infection rates; inform alert programs; and improve performance, strategies, and competence development [13, 14]. The distribution of HAIs between hospitals and hospital wards can have significant variability; therefore, it is essential to create specific reports for healthcare settings that are under investigation to reduce bias and provide dynamic monitoring to allow proper countermeasures to be taken for the prevention and management of HAIs. EHRs are an invaluable source of information; however, most of the time, information in EHRs are stored in separate databases that are not networked.

Despite the numerous studies concerning prevalence and incidence of HAIs, and their correlation with specific risk factors, surprisingly fewer data are available on the integration of HDFs and Microbiology laboratory data. This approach is particularly useful to identify not only the correlation between risk factors associated with HAIs (both socio-demographic and hospital related), but also to evaluate their appropriate coding in the HDFs, obviously associated with the economic losses for the hospital. This retrospective observational study re-elaborates the laboratory data retrieved from different EHRs of the university hospital “Policlinico Tor Vergata” (PTV) of Rome, Italy, and integrates them with hospital discharge forms (HDFs). We suggest a novel approach for monitoring circulating alert microorganisms and for estimating the annual number of HAIs by offering a methodological tool to investigate the epidemiological aspects and possible cost implications.

## Materials and methods

The conducted retrospective observational study analyzed ordinary inpatient admissions at the PTV hospital in Rome. All patients discharged from the medical and surgical wards from January 1 to December 31, 2018, were included in the analysis. Data from 2020 to 2021 have been excluded from our investigation due to possible bias introduced by the emergency situation caused by COVID-19. Moreover, the comparison with data form 2019 will be the object of further studies.

Data extraction was performed by analyzing HDFs in the databases of the Hospital Information System. These data were compiled using the AREAS-ADT information system and the ICD-9-CM classification (2007 version, which is the current standard in Italian hospitals). This newly assembled database was further processed to evaluate the quantitative and qualitative variables and the produced diagnostic-related groups (DRGs). The results of research requests for HAI and AMR evaluations were extracted from the database of the Complex Operational Unit of Microbiology.

### Record linkage

First, the database was “cleaned” for individual fields of records related to personal data (patient’s name and date of birth) to achieve a semideterministic agreement between laboratory databases by using the type of sample categorized by body district of origin as keys (in addition to personal data) to identify suspected infections related to care assistance (ICAs). Second, on the basis of the patient identification number used as a deterministic key, a unique single database was created. This database shows the flow of 2018 DPR first admissions, which are linked to information of any rehospitalization and samples of alert microorganisms divided by type of infection.

### Inclusion and exclusion criteria

Ordinary admissions (cod.1 variable “admission regime”) from all operating units of PTV discharged before December 31, 2018, were included in the analysis. Admissions in the nonordinary regimen (code 2-Day Hospital, code 3-Home treatment, code 4-Day Surgery with overnight stay) and patients < 18 years old were excluded because the hospital does not have a pediatric ward.

Regarding the analysis of bacteriological data, the exclusion criteria included findings of contaminants and diagnoses of microbiological infection occurring 24 hours after hospitalization (in accordance with the definition of HAIs). In the specific case of *Clostridioides difficile*, which is linked to the intrahospital administration of antibiotics, samples taken during the first seven days of hospitalization were also excluded. For each detected microorganism, the dates of the first and last detection before discharge were reported.

## Variables investigated

The following variables were collected from the HDFs:

- Personal data (age, sex, fiscal code, etc.)
- Main diagnosis (DPR) and secondary diagnoses (first to fifth) at discharge
- Codes related to procedures performed during hospitalization
- Length of stay (LOS)
- Type of discharge
- Educational qualification: low educational attainment (lower secondary school, elementary school, or no degree [ISCED 0–2]), medium educational attainment (upper secondary school [ISCED 3–4]), and high educational attainment (bachelor’s degree or other tertiary degree [ISCED 5–8])
- Nationality
- Marital status

The isolated HAI microorganisms in our sample can be classified into 56 families and more than 150 species. For this study, we focused our analysis on the eight most represented microorganisms in our sample, namely, *A*. *baumannii*, *Escherichia coli*, *K*. *pneumoniae*, *P*. *aeruginosa*, *Clostridioides difficile*, *Candida* spp., *Enterococcus* spp., and *Staphylococcus* spp.

The following variables were retrieved from the laboratory database:

- Positive sample of the HAI microorganism with date of collection and department where the request was sent
- Type of collected sample
- Positivity of the tested AMR phenotype (MDR *A*. *baumannii*, extended-spectrum beta-lactamase *Escherichia coli*, *K*. *pneumoniae* with at least one AMR profile, and *Staphylococcus* spp with at least one AMR profile)

All data was processed to create index variables for statistics.

### Statistical analysis

Data were anonymized before conducting the statistical analyses. Comparisons were performed using a two-sample t-test or Pearson’s chi-squared test as appropriate. After performing descriptive analysis (frequency, histograms, means, medians IQ, SD, etc.), univariate and multivariate logistic regression models were used to calculate the risk and estimate associations. Statistical analyses were performed using SPSS v.26.0 (IBM Corp., Armonk, NY, USA).

### Ethics

This project was conducted in accordance with the Declaration of Helsinki. The project received approval from the Independent Ethics Committee of the University Hospital PTV, Rome, Italy (2022 –Protocol number 66.22) that waived the need for informed consent. To meet privacy requirements, data were anonymized by entering them in an Excel database protected by an encrypted key, which is made available only to the investigators involved in the study.

## Results

**Fig 1:**
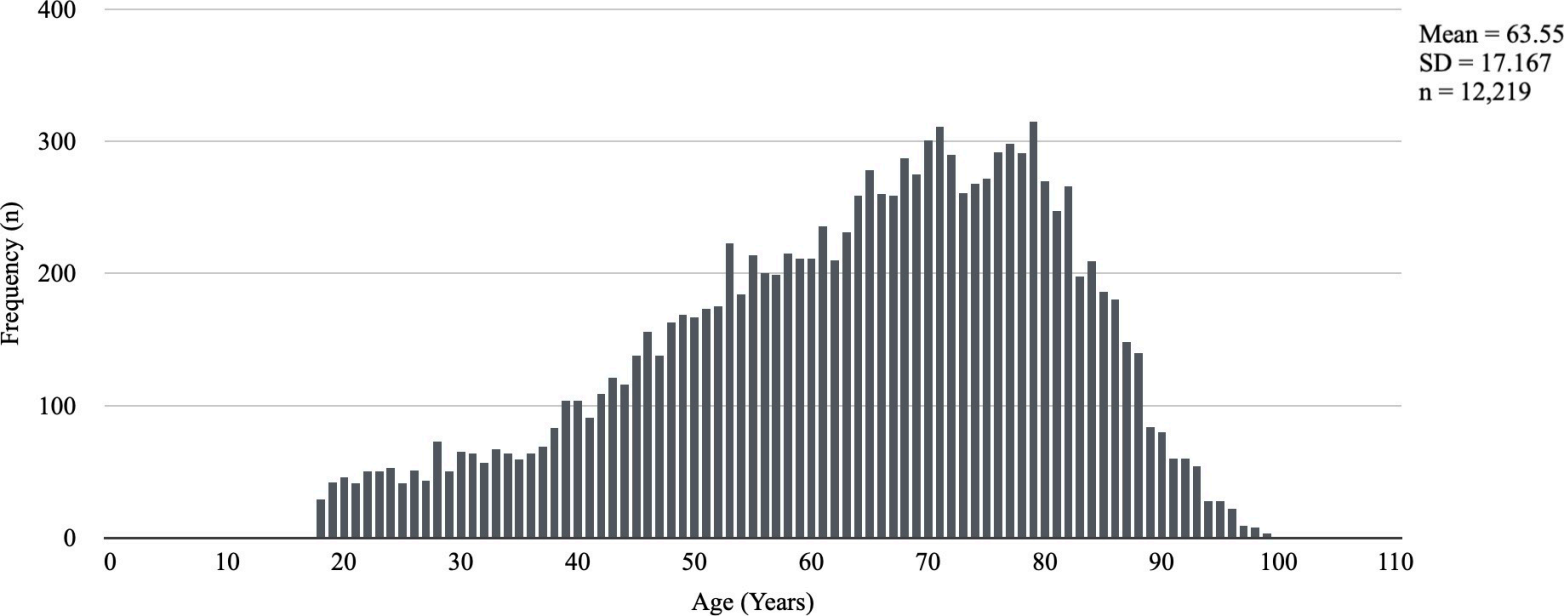
Age distribution in the hospitalized sample.

**Fig 2:**
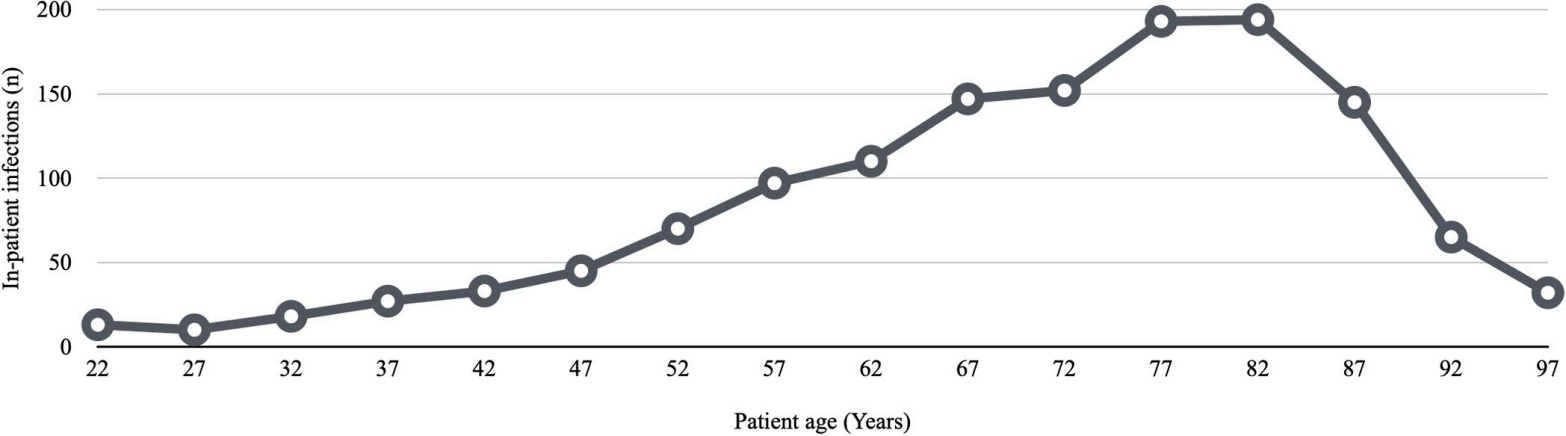
Age distribution of inpatient infections.

The study sample included 12,219 individuals: 6,770 were males (55.4%), and 5,449 were females (44.6%). The mean age of the patients was 63.6 ± 17.17 years (Fig 1). A total of 6,541 individuals (53.5%) were ≥ 65 years old. The average hospital stay was 9.4 ± 13.63 days, with a median of 5 days. Italian and non-Italian were the nationalities in 91.8% (11,222 individuals) and 8.2% (997 individuals) of cases, respectively. Educational level was low [15] in 76.6% of cases (9,354 individuals) and high in 23.4% of cases (2,865 individuals). The proportion of married individuals was 75.2% (9,183). A total of 1,654 individuals (13.5%) were admitted twice. Table 1 shows the data related to the descriptive analysis of the sample. Figs 1 and 2 respectively show the age distribution of the sample and inpatient infections.

**Table 1:** Descriptive analysis of the samples (n = 12,219).

Investigated variable	N	%	Mean	SD
**Age**			63.55	17.17
Range 18–102 years				
**Gender**				
Male	6,770	55.40		
Female	5,449	44.60		
**Education level**				
None	82	0.70		
Elementary school	1,497	12.30		
Lower secondary school	7,775	63.60		
Upper secondary school	2,312	18.90		
Bachelor’s degree/tertiary degree	553	4.50		
**Marital status**				
Single	3,036	24.80		
Married	9,183	75.20		
**Nationality**				
Italian	11,222	91.80		
Others	997	8.20		
**LOS**			9.40	13.63
Range 0–360 days				
**Rehospitalization**				
No	10,565	86.50		
Yes	1,654	13.50		

The mean age was 68.3 ± 15.771 years in the infection group and 63.0 ± 17.243 years in the noninfection group (t = 10.781; p < 0.001). The mean LOS was 31.03 ± 25.610 days in the infection group and 6.71 ± 7.865 days in the noninfection group (t = 74.683; p < 0.001).

**Fig 3:**
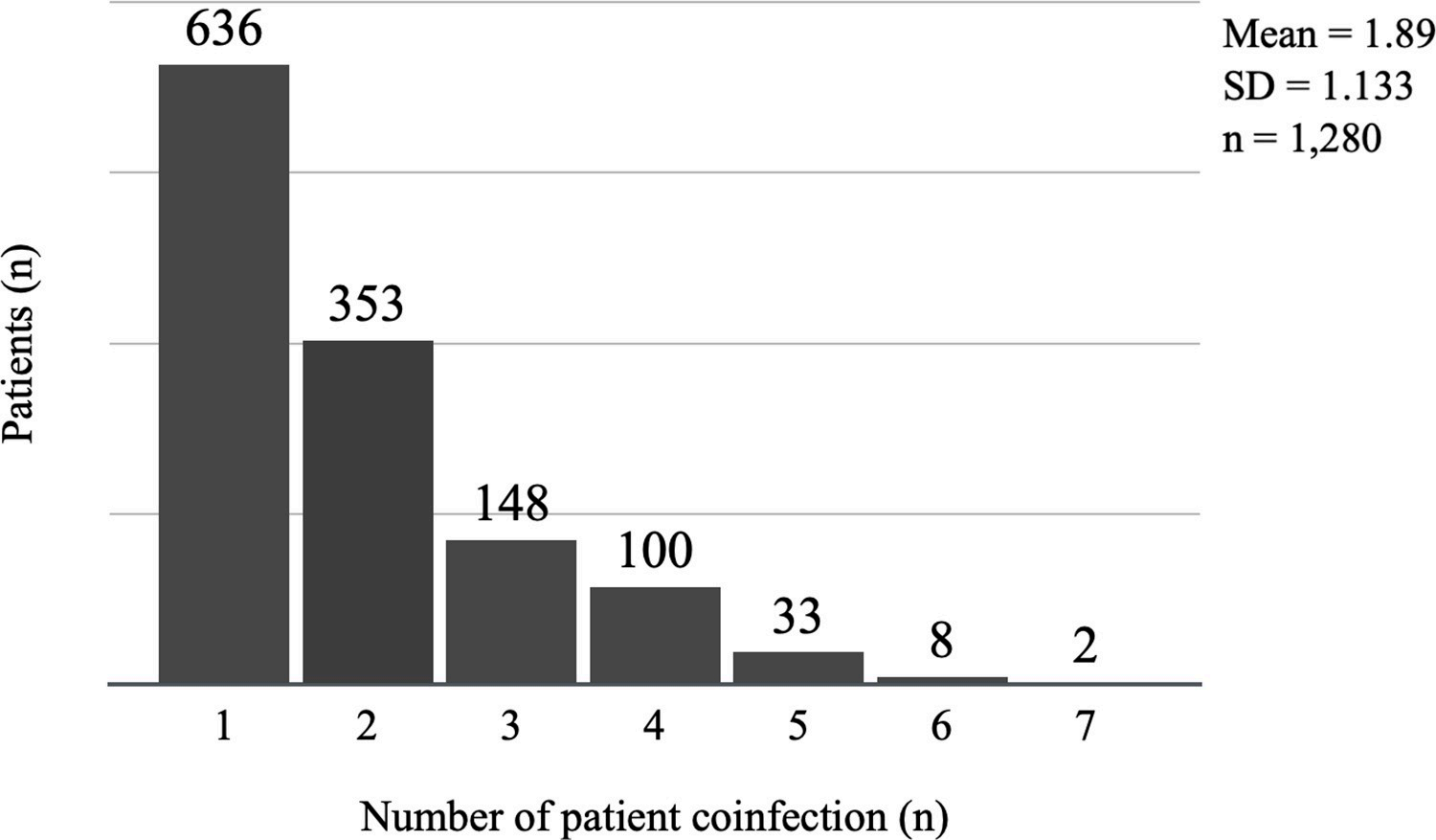
Distribution of concurrent infections on the sample.

In this retrospective analysis, 1,352 individuals (11.1%) presented one or more suspected ICAs. The number of reported HAI microorganisms (including *Candida* spp., *Proteus*, *Aspergillus* spp.) was 3,549, thus resulting in an average of 2.62 microorganism reported per positive patient. Among these, 2,938 microorganisms (67.6%) are regarded alert microorganisms for AMR, thus resulting in an average of 1.77 AMR alert microorganism reported per positive patient. Concurrent infections from different microorganisms are very frequent, and approximately 50% of infected individuals tested positive for more than one microorganism during their period of hospitalisation (Fig 3).

Univariate analysis for the risk of infection showed statistically significant associations with age, LOS, marital status, and low level of education (Table 2). However, there was no significant association between sex and nationality. The binary logistic regression approach was used for multivariate analysis, which showed a persistent association of HAIs with age and LOS in the hospital (Table 2).

**Table 2:** Association between sociodemographic and outcome variables for infections with HAI associated microorganisms: a binary logistic regression analysis.

	Univariate analysis				Multivariate analysis forward stepwise method			
Investigated variable	OR	95% CI		p	OR	95% CI		p
		lower	upper			lower	upper	
Age (decade)	**1.217**	1.174	1.262	<0.001	**1.178**	1.126	1.233	<0.001
Gender (male)	0.980	0.875	1.098	= 0.731				
Education level	**0.766**	0.705	0.832	<0.001				
Marital status (single)	**1.361**	1.184	1.566	<0.001				
Nationality (italian)	0.835	0.670	1.040	= 0.107				
LOS (days)	**1.146**	1.138	1.153	<0.001	**1.144**	1.137	1.152	<0.001

Concerning wards, the analysis showed a higher risk of infection associated with medical intervention than with surgical intervention (OR = 1.603; 95% CI = 1.430–1.797; p < 0.001). The risk increased among those over the age of 65 (OR = 1.872; 95% CI = 1.616–2.169; p<0.001), whereas the risk was not statistically significant among those under the age of 65 (OR = 1.185; 95% CI = 0.984–1.428; p = 0.074). Table 3 presents the frequency distributions.

**Table 3:** Distribution of HAI microorganisms by ward type.

	Inpatient ward	Presence of infection		Total
		n	%	
All patients	Medical	780	13.5%	5,775
	Surgical	572	8.9%	6,444
	**Total**	**1,352**	**11.1%**	12,219
Patients with age **≥ 65 years**	Medical	544	16.9%	3,226
	Surgical	324	9.8%	3,315
	**Total**	**868**	**13.3%**	6,541
Patients with age **< 65 years**	Medical	236	9.3%	2,549
	Surgical	248	7.9%	3,129
	**Total**	**484**	**8.5%**	5,678

ICUs had a higher risk of infection in all patients (OR = 3.071; 95% CI = 2.539–3.715; p<0.001). Considering age, the risk for those under 65 was higher (OR = 5.478; 95% IC = 4.036–7.435; p < 0.001) than for those over 65 (OR = 2.094; 95% CI = 1.639–2.675; p < 0.001).

Table 4 lists the frequency of infections per site. Sociodemographic and outcome variables were analyzed for the eight microorganisms under investigation and are presented in Table 5.

**Table 4:** Frequency of alert microorganisms over the site of infection.

	Bacteremia / sepsis		Respiratory tract		Gastrointestinal tract		Urinary tract		Surgical site		Other sites	
	n	%	n	%	n	%	n	%	n	%	n	%
*Staphylococcus* spp	473	50.2	95	16.4	0	0.0	16	1.6	66	14.7	95	21.3
*Klebsiella pneumoniae*	98	10.4	65	11.2	41	39.4	97	9.6	45	10.0	20	4.5
*Enterococcus* spp	98	10.4	29	5.0	0	0.0	243	24.1	122	27.2	81	18.1
*Candida* spp	68	7.2	93	16.1	2	1.9	305	30.3	47	10.5	96	21.5
*Escherichia coli*	47	5.0	30	5.2	3	2.9	169	16.8	41	9.2	30	6.7
*Acinetobacter baumannii*	33	3.5	59	10.2	22	21.2	26	2.6	25	5.6	19	4.3
*Pseudomonas aeruginosa*	21	2.2	69	12.0	1	1.0	56	5.6	29	6.5	22	4.9
*Clostridioides difficile*	0	0.0	0	0.0	33	31.7	0	0.0	0	0.0	0	0.0
*Vibrions*	0	0.0	0	0.0	1	1.0	0	0.0	0	0.0	0	0.0
*Proteus*	0	0.0	0	0.0	0	0.0	33	3.3	29	6.5	0	0.0
*Aspergillus*	0	0.0	0	0.0	0	0.0	0	0.0	0	0.0	18	4.0
Others	105	11.1	138	23.9	1	0.8	63	6.3	44	9.8	66	14.8
**Total**	**943**	**100.0**	**578**	**100.0**	**104**	**100.0**	**1,008**	**100.0**	**448**	**100.0**	**447**	**100.0**

**Table 5:** Intrahospital decease and infections: a binary logistic regression analysis.

	Univariate Analysis				Multivariate analysis forward stepwise method			
Investigated variable	OR	95% CI		p	OR	95% CI				p
		lower	upper			lower	upper	
*Staphylococcus* spp	**7.801**	6.306	9.651	<0.001	**2.948**	2.249	3.864	<0.001
*Pseudomonas aeruginosa*	**9.106**	6.584	12.595	<0.001	**2.435**	1.648	3.600	<0.001
*Klebsiella pneumoniae*	**9.334**	7.084	12.299	<0.001	**2.640**	1.884	3.700	<0.001
*Escherichia coli*	**3.108**	2.203	4.384	<0.001				
*Enterococcus* spp	**4.835**	3.797	6.157	<0.001				
*Clostridioides difficile*	**9.748**	4.828	19.681	<0.001	**4.841**	2.164	10.830	<0.001
*Candida spp*	**8.589**	6.891	10.707	<0.001	**3.131**	2.368	4.141	<0.001
*Acinetobacter baumannii*	**12.558**	8.816	17.889	<0.001	**2.443**	1.585	3.764	<0.001

A binary logistic regression analysis was conducted to provide a risk calculation between infections and intrahospital death that accounted for 26% (278/1,352) of patients with at least one HAI in our study. Table 5 shows the results. All reported microorganisms were significantly associated with an increased risk of mortality in the univariable analysis, and this association was confirmed in the multivariate analysis, except for *Escherichia coli* and *Enterococcus* spp. Multivariate analysis was probably influenced by the high rate of concurrent infections.

Regarding the antibiotic resistance spectra, all isolated microorganisms were tested for resistance phenotypes. Overall, 141 patients showed at least 1 infection with AMR phenotype; for the 4 AMR microorganisms considered in this study, we first evaluated the association with intrahospital death and then evaluated the effect of these microorganisms when considering the mortality associated with an infection caused by the same microorganism without an AMR profile (Table 6).

**Table 6:** Univariate analysis for intrahospital decease associated with AMR infections and non-AMR infections.

	Non-AMR						AMR				
Investigated variable	OR	95% CI		p	n	OR		95% CI		p	n
		lower	upper					lower	upper		
*Staphylococcus* spp	**6.563**	5.197	8.287	<0.001	676	**18.275**		11.355	29.412	<0.001	70
*Klebsiella pneumoniae*	**8.282**	6.022	11.391	<0.001	303	**12.539**		7.520	20.908	<0.001	63
*Escherichia coli*	**2.899**	2.013	4.176	<0.001	310	**6.171**		2.441	15.600	<0.001	9
*Acinetobacter baumannii*	**9.699**	6.457	14.571	<0.001	156	**33.997**		15.627	73.963	<0.001	28

In addition, we evaluated the percentage of HAIs for which coding was missing from the DRGs. In 407 cases (30.1%), coding for the diagnosis of infection by HAIs was not performed. Coding in medical wards, surgical wards, and ICUs was lacking in 27.0%, 36.8%, and 26.4% of cases, respectively (Table 7).

**Table 7:** Share of infection coding performed by ward type.

Ward	Coding not done		Total	
	n	%	n	%
Medical	203	27.0%	753	100.0%
Surgical	162	36.8%	440	100.0%
Intensive care unit	42	26.4%	159	100.0%
**Total**	**407**	**30.1%**	**1,352**	**100.0%**

## Discussion

The results of this study confirm the alarming data described in the literature regarding the spread of microorganisms of concern and consequent infections in acute care institutions.

Our analysis investigated a specific case study, that of PTV, a university hospital that serves as a referral for high medical specialty functions for about 1,5 million citizens living in the city of Rome and province. Our sample showed a prevalence of infections, in ordinary hospitalization, of 11.1% (1352/12219). This data is high in comparison with the most recent available data from studies across Europe [1, 16] and Italy [17] which showed a prevalence of HAIs of approximately 8.00%.

As HAIs are a multifactorial phenomenon, our study allowed us to investigate in further detail their incidence in relation to several factors both sociodemographic and associated with specific medical procedures. Moreover, while many studies focus on the incidence of infections due to a specific microorganism, our approach allowed us to investigate the phenomenon as a whole, showing interesting results especially concerning multiple HAIs acquired by the single patient. The combined approach of data from the laboratory, the clinical wards and from hospital discharge card allowed a broader view on the topic, and gave more interesting insight. First, the sociodemographic variables associated with HAIs provide us important insights, thus highlighting their role in defining the absolute risk of developing a HAIs (Table 2). Our data showed that being unmarried increased the risk of HAIs. We inferred that marital status could be an indirect indication of loneliness, which is a powerful risk factor for infectious disease susceptibility [18, 19] and immunosuppression (i.e. higher susceptibility of developing HAIs).On the other hand a low education level is associated with poor socioeconomic conditions linked to physical weakness and comorbidities [20].

Nevertheless, the association of the education level with HAI was not confirmed on the multivariate analysis.

In our study, both in univariate and multivariate analysis, age and LOS are significantly associated with HAIs, while gender is not associated in either analysis, this is supported also by the study of Golfera and colleagues [21]. However, as demonstrated in the narrative revision of Cristina and colleagues (2021) elderly patients are identified as belonging to the high-risk group for the development of healthcare-associated infections (HAI) due to the age-related immune system decline, known as immunosenescence [22].

The association of the above-mentioned variables with HAI was not confirmed on the multivariate analysis, probably because of the effect of age. We confirmed the well-known association with the increase in LOS appears in our analysis. Ohannessian and colleagues [23] estimated the change in LOS due to infection(s); by using a multistate model and the time of infection onset. Results from their study show an increase in LOS of 5.0 days (95% CI = 4.6–5.4 days). The LOS increased with the number of infected sites and was higher for patients who were discharged alive from the ICU, but no increase in LOS was found for patients presenting with late-onset HAI, after day 25 of admission [23]. This suggests that the increase in LOS is attributable to infection at the early stage of hospitalization. However in the study of Cristina and colleagues HAI in geriatric patients are responsible for longer hospital stays [22].

Concerning wards, our study showed that, the risk of HAI is higher in medical wards than surgical wards (13.5% and 8.9% of patients, respectively) (Table 3) this is also confirmed by the literature [21, 24]. Our study confirmed this data for both over 65 and under 65 year old patients. This can be explained by the greater number of elderly patients (>65 years) in medical wards than in surgical wards; however, the difference in HAI incidence was not confirmed only considering patients under 65 years. We can therefore assume that perioperative prophylaxis in surgical wards plays a role in reducing infections [25] even if, particular attention must be paid to compliance with the protocol and to its duration to avoid the promotion of AMR insurgence [16, 26, 27].

Concerning the site of infection, HAIs that are frequently observed in our study include urinary tract infections and BSIs; this fact is in accordance with the global trends described in the literature [6, 7, 28].

Intra-hospital death is frequently observed in patients with HAIs, and all analysed microorganisms are significantly associated with increased risk of mortality in the univariate analysis. In multivariate analysis there is no association with increased risk of mortality and Escherichia *coli* and *Enterococcus spp* microorganisms.

In this study we showed that approximately 50% of patients who acquired HAIs during their hospitalization tested positive for at least two or more microorganisms, therefore the multivariate analysis provided relevant insights into the effect of concurrent infections. The multivariate analysis showed that some microorganisms, such as *Clostridioides difficile*, occur less frequently than others. On the other hand, microorganisms such as *Escherichia coli* and *Enterococcus spp*. are more likely associated with at least one concurrent HAI, this finding probably explains why they are not associated with mortality in the multivariate analysis (Table 5).

The same approach has been used for infections caused by AMR microorganisms. The risk of intrahospital death is extremely high compared to infections from the same microorganisms with no AMR profile. A relevant example is MDR *Acinetobater baumannii* which increases mortality by 34 times, compared with 9 times in non-AMR cases (Table 6).

## Conclusions

The phenomenon investigated represents a major challenge for public health worldwide, but few epidemiological studies have been conducted on the subject. Furthermore, epidemiological studies are usually conducted in limited areas and with only partial integration of laboratory data, often leading to the detriment of an overall picture.

The record-linkage experiment between laboratory data and HDF flows represents an innovative method from the epidemiological point of view in the assessment of HAIs, which, to the best of our knowledge, has never been fully used, particularly in situations where an integrated regional bacteriological data collection system has not been implemented.

The author considers this approach as a promising tool for deciphering the epidemiological frame of intrinsic comorbidity and is a starting point in combining environmental, clinical, social individual and organizational factors to calculate and define the risk of HAIs.

The proportion of preventable HAIs is high, and infections associated with certain procedures can be prevented by reducing unnecessary interventions, choosing safer equipment, adopting aseptic patient care measures, and observing proper hand washing techniques [12–14]. The cost of HAIs is high to the patient and the facility in both health and economic terms; hence, there is a need to adopt safe care practices that prevent or control transmission, particularly of alert microorganisms. Control programs need to be implemented at different levels (national, regional, and local) to ensure the implementation of measures that have proven effective in minimizing the risk of infections, but this cannot be achieved without improving epidemiological knowledge by conducting prospective studies and improving the transmission of information in administrative flows.

### Limitations

The weaknesses of this study includes its lack of systematic data collection and record linkage with environmental monitoring (surfaces, fomites, air, water, medical devices, etc.) and medical surgical procedures/devices. We are currently working to integrate these types of data into the system. In addition, we need to integrate key data from personnel attitudes regarding hand hygiene and other behaviours in daily activities. Microbiological data is integrated in our database but only for patients who tested positive for HAIs, thus resulting in the lack of information about the total number of microbiological tests conducted. Some variables associated with HAIs, except LOS in hospitals, had an unclear causal relationship with the risk of infection because of the observational nature of the study. A prospective study could be useful for assessing this bidirectional relationship.

## References

[1] Suetens C, Latour K, Kärki T, Ricchizzi E, Kinross P, Moro ML, et al. Prevalence of healthcare-associated infections, estimated incidence and composite antimicrobial resistance index in acute care hospitals and long-term care facilities: results from two European point prevalence surveys, 2016 to 2017. Eurosurveillance [Internet]. 2018 Nov 15 [cited 2021 Nov 15];23(46). Available from: https://www.eurosurveillance.org/content/10.2807/1560-7917.ES.2018.23.46.1800516 30458912 10.2807/1560-7917.ES.2018.23.46.1800516 PMC6247459

[2] Bordino V, Vicentini C, D’Ambrosio A, Quattrocolo F, Zotti CM, Novati R, et al. Burden of healthcare-associated infections in Italy: incidence, attributable mortality and disability-adjusted life years (DALYs) from a nationwide study, 2016. J Hosp Infect. 2021 Jul;113:164–71. doi: 10.1016/j.jhin.2021.04.023 33940090

[3] Monasta L, Abbafati C, Logroscino G, Remuzzi G, Perico N, Bikbov B, et al. Italy’s health performance, 1990–2017: findings from the Global Burden of Disease Study 2017. Lancet Public Health. 2019 Dec;4(12):e645–57. doi: 10.1016/S2468-2667(19)30189-6 31759893 PMC7098474

[4] Epicentro. Italy’s antimicrobial resistance surveillance system (AR-ISS) report dati 2019; 2020 [cited 2022 Feb 22]. Database: Archivio dei rapporti delle sorveglianze AR-ISS e CPE [internet].Available from:: https://www.epicentro.iss.it/antibiotico-resistenza/ar-iss/RIS-1_2020.pdf.

[5] European Centre for Disease Prevention and Control. Country summaries antimicrobial resistance in the EU/EEA (EARS-Net) Annual Epidemiological Report 2019; 2020 [cited 2022 Feb 22]. Database: ECDC [internet]. Available from: https://www.ecdc.europa.eu/sites/default/files/documents/Country%20summaries-AER-EARS-Net%20202019.pdf.

[6] Liu JY, Dickter JK. Nosocomial Infections. Gastrointest Endosc Clin N Am. 2020 Oct;30(4):637–52. 32891222 10.1016/j.giec.2020.06.001

[7] Stewart S, Robertson C, Pan J, Kennedy S, Dancer S, Haahr L, et al. Epidemiology of healthcare-associated infection reported from a hospital-wide incidence study: considerations for infection prevention and control planning. J Hosp Infect. 2021 Aug;114:10–22. doi: 10.1016/j.jhin.2021.03.031 34301392

[8] Deng Y, Zheng Z, Cheng S, Lin Y, Wang D, Yin P, et al. The factors associated with nosocomial infection in elderly hip fracture patients: gender, age, and comorbidity. Int Orthop. 2021 Dec;45(12):3201–9. doi: 10.1007/s00264-021-05104-3 34350473

[9] O’Toole RF. The interface between COVID-19 and bacterial healthcare-associated infections. Clin Microbiol Infect. 2021 Jun;S1198743X21002974. doi: 10.1016/j.cmi.2021.06.001 34111586 PMC8182977

[10] Cantón R, Gijón D, Ruiz-Garbajosa P. Antimicrobial resistance in ICUs: an update in the light of the COVID-19 pandemic. Curr Opin Crit Care. 2020 Oct;26(5):433–41. doi: 10.1097/MCC.0000000000000755 32739970

[11] Afshinnekoo E, Bhattacharya C, Burguete-García A, Castro-Nallar E, Deng Y, Desnues C, et al. COVID-19 drug practices risk antimicrobial resistance evolution. Lancet Microbe. 2021 Apr;2(4):e135–6. doi: 10.1016/S2666-5247(21)00039-2 33655229 PMC7906697

[12] Haque M, McKimm J, Sartelli M, Dhingra S, Labricciosa FM, Islam S, et al. Strategies to Prevent Healthcare-Associated Infections: A Narrative Overview. Risk Manag Healthc Policy. 2020 Sep;Volume 13:1765–80. doi: 10.2147/RMHP.S269315 33061710 PMC7532064

[13] Ridelberg M, Nilsen P. Using surveillance data to reduce healthcare–associated infection: a qualitative study in Sweden. J Infect Prev. 2015 Sep;16(5):208–14. doi: 10.1177/1757177415588380 28989432 PMC5074157

[14] de Bruin JS, Seeling W, Schuh C. Data use and effectiveness in electronic surveillance of healthcare associated infections in the 21st century: a systematic review. J Am Med Inform Assoc. 2014 Sep 1;21(5):942–51. doi: 10.1136/amiajnl-2013-002089 24421290 PMC4147601

[15] UNESCO Institute for Statistics. International standard classification of education: ISCED 2011 [Internet]. Montreal, Quebec: UNESCO Institute for Statistics; 2012 [cited 2021 Nov 15]. Available from: http://www.uis.unesco.org/Education/Documents/isced-2011-en.pdf

[16] Cassini A, Plachouras D, Eckmanns T, Abu Sin M, Blank HP, Ducomble T, et al. Burden of Six Healthcare-Associated Infections on European Population Health: Estimating Incidence-Based Disability-Adjusted Life Years through a Population Prevalence-Based Modelling Study. Harbarth S, editor. PLOS Med. 2016 Oct 18;13(10):e1002150. doi: 10.1371/journal.pmed.1002150 27755545 PMC5068791

[17] Italian ministry of health. Studio di prevalenza italiano sulle infezioni correlate all’assistenza e sull’uso di antibiotici negli ospedali per acuti—protocollo ECDC [internet]. Università di Torino; 2018 [cited 2022 Feb 22]. Available from: https://www.salute.gov.it/imgs/C_17_pubblicazioni_2791_allegato.pdf.

[18] Liotta G, Marazzi MC, Orlando S, Palombi L. Is social connectedness a risk factor for the spreading of COVID-19 among older adults? The Italian paradox. Abete P, editor. PLOS ONE. 2020 May 21;15(5):e0233329. doi: 10.1371/journal.pone.0233329 32437377 PMC7241742

[19] Yanguas J, Pinazo-Henandis S, Tarazona-Santabalbina FJ. The complexity of loneliness. Acta Bio Medica Atenei Parm. 2018 Jun 7;89(2):302–14. doi: 10.23750/abm.v89i2.7404 29957768 PMC6179015

[20] Donnelly JP, Lakkur S, Judd SE, Levitan EB, Griffin R, Howard G, et al. Association of Neighborhood Socioeconomic Status With Risk of Infection and Sepsis. Clin Infect Dis. 2018 Jun 1;66(12):1940–7. doi: 10.1093/cid/cix1109 29444225 PMC6248765

[21] Golfera M, Toscano F, Cevenini G, Marco MFD, Porchia BR, Serafini A, et al. Predicting Healthcare-associated Infections: are Point of Prevalence Surveys data useful? J Prev Med Hyg. 2022 Jun;63(2):E304–9. doi: 10.15167/2421-4248/jpmh2022.63.2.1496 35968075 PMC9351422

[22] Cristina ML, Spagnolo AM, Giribone L, Demartini A, Sartini M. Epidemiology and Prevention of Healthcare-Associated Infections in Geriatric Patients: A Narrative Review. Int J Environ Res Public Health. 2021 May;18(10):5333. doi: 10.3390/ijerph18105333 34067797 PMC8156303

[23] Ohannessian R, Gustin MP, Bénet T, Gerbier-Colomban S, Girard R, Argaud L, et al. Estimation of Extra Length of Stay Attributable to Hospital-Acquired Infections in Adult ICUs Using a Time-Dependent Multistate Model*: Crit Care Med. 2018 Jul;46(7):1093–8. doi: 10.1097/CCM.0000000000003131 29642107

[24] Chernet AZ, Dasta K, Belachew F, Zewdu B, Melese M, Ali MM. Burden of Healthcare-Associated Infections and Associated Risk Factors at Adama Hospital Medical College, Adama, Oromia, Ethiopia. Drug Healthc Patient Saf. 2020 Oct;Volume 12:177–85. doi: 10.2147/DHPS.S251827 33116913 PMC7569037

[25] Prospero E, Barbadoro P, Marigliano A, Martini E, D’Errico MM. Perioperative antibiotic prophylaxis: improved compliance and impact on infection rates. Epidemiol Infect. 2011 Sep;139(9):1326–31. doi: 10.1017/S0950268810002505 21087536

[26] Cicuttin E, Sartelli M, Scozzafava E, Tartaglia D, Cremonini C, Brevi B, et al. Antibiotic Prophylaxis in Torso, Maxillofacial, and Skin Traumatic Lesions: A Systematic Review of Recent Evidence. Antibiotics. 2022 Jan 21;11(2):139. doi: 10.3390/antibiotics11020139 35203743 PMC8868174

[27] Rothe, Kathrin M.D.; Münster, Nathalie; Hapfelmeier, Alexander Ph.D.; Ihbe-Heffinger, Angela Ph.D.; Paepke, Stefan M.D.; Niemeyer, Markus M.D., Ph.D.; et al., Plastic and Reconstructive Surgery: January 31, 2022—Volume—Issue—10.1097/PRS.0000000000008900 doi: 10.1097/PRS.0000000000008900 Does the Duration of Perioperative Antibiotic Prophylaxis Influence the Incidence of Postoperative Surgical-Site Infections in Implant-Based Breast Reconstruction in Women with Breast Cancer? A Retrospective Study. 35103626

[28] Kollef, Marin H. MD1; Torres, Antoni MD, PhD2; Shorr, Andrew F. MD, MPH, MBA3; Martin-Loeches, Ignacio MD, PhD4; Micek, Scott T. PharmD. Nosocomial Infection, Critical Care Medicine. 10.1097/CCM.0000000000004783 33438970

